# Case Report: Malignant peripheral nerve sheath tumor of the thoracic spine with postoperative recurrence and intracranial metastasis—a review of the literature

**DOI:** 10.3389/fonc.2025.1640238

**Published:** 2025-10-17

**Authors:** He-Lu Wang, Hong-Tao Zhang

**Affiliations:** ^1^ School of Clinical Medicine, Shandong Second Medical University, Weifang, China; ^2^ Neurosurgery Department, Yantai Yuhuangding Hospital, Yantai, China

**Keywords:** malignant peripheral nerve sheath tumor, intradural tumor, perineural invasion, Enneking-appropriate resection, EA resection, neuroaxis dissemination

## Abstract

Malignant peripheral nerve sheath tumors (MPNSTs) are uncommon and biologically aggressive sarcomas. Although primary involvement of the thoracic spine is rare, the occurrence of intracranial metastasis typically indicates advanced-stage disease and portends a poor prognosis. We report a unique case of a 64-year-old man with a primary thoracic spinal MPNST that recurred three months after surgery and developed intracranial metastasis within two months. Initial imaging revealed a subdural lesion at the T11–T12 level. Postoperative histopathology confirmed MPNST with a Ki-67 index of 50%. Shortly after surgery, the tumor recurred locally and metastasized to the left parietal lobe. Histological examination of the intracranial lesion confirmed metastatic MPNST. This case highlights the potential for MPNST to metastasize to the brain via atypical routes, underscoring the importance of heightened clinical vigilance and the need for multimodal strategies to detect brain metastasis at an early stage.

## 1 Introduction

Malignant peripheral nerve sheath tumor (MPNST) is a rare and highly aggressive sarcoma, comprising 3%–5% of all soft tissue sarcomas. The five-year survival rate remains extremely low, reflecting its high propensity for local recurrence and distant metastasis. Only 4%–5% of cases are associated with neurofibromatosis type 1 (NF1), while the majority are sporadic or secondary to prior radiation exposure. Primary MPNSTs originating within the spinal canal are exceptionally rare, and intracranial metastases from such lesions are even more uncommon. Here, we report a rapidly progressive case of sporadic thoracic intradural MPNST with early postoperative recurrence and leptomeningeal intracranial spread. This case highlights critical diagnostic challenges, the limitations of conventional imaging and pathology, and the urgent need for refined surgical and molecular strategies in managing this rare malignancy.

## 2 Case report

A 64-year-old male presented with a one-year history of progressive muscle atrophy in the right lower limb and diminished exercise tolerance. His symptoms included difficulty ascending stairs and numbness in the right plantar region. Over the preceding three months, his condition deteriorated, manifesting as symmetric atrophy of both lower limbs, accompanied by intermittent, self-resolving muscle spasms lasting 5–10 minutes. On August 2, 2023, he was admitted to our institution’s Department of Neurology.

**Figure 1:**
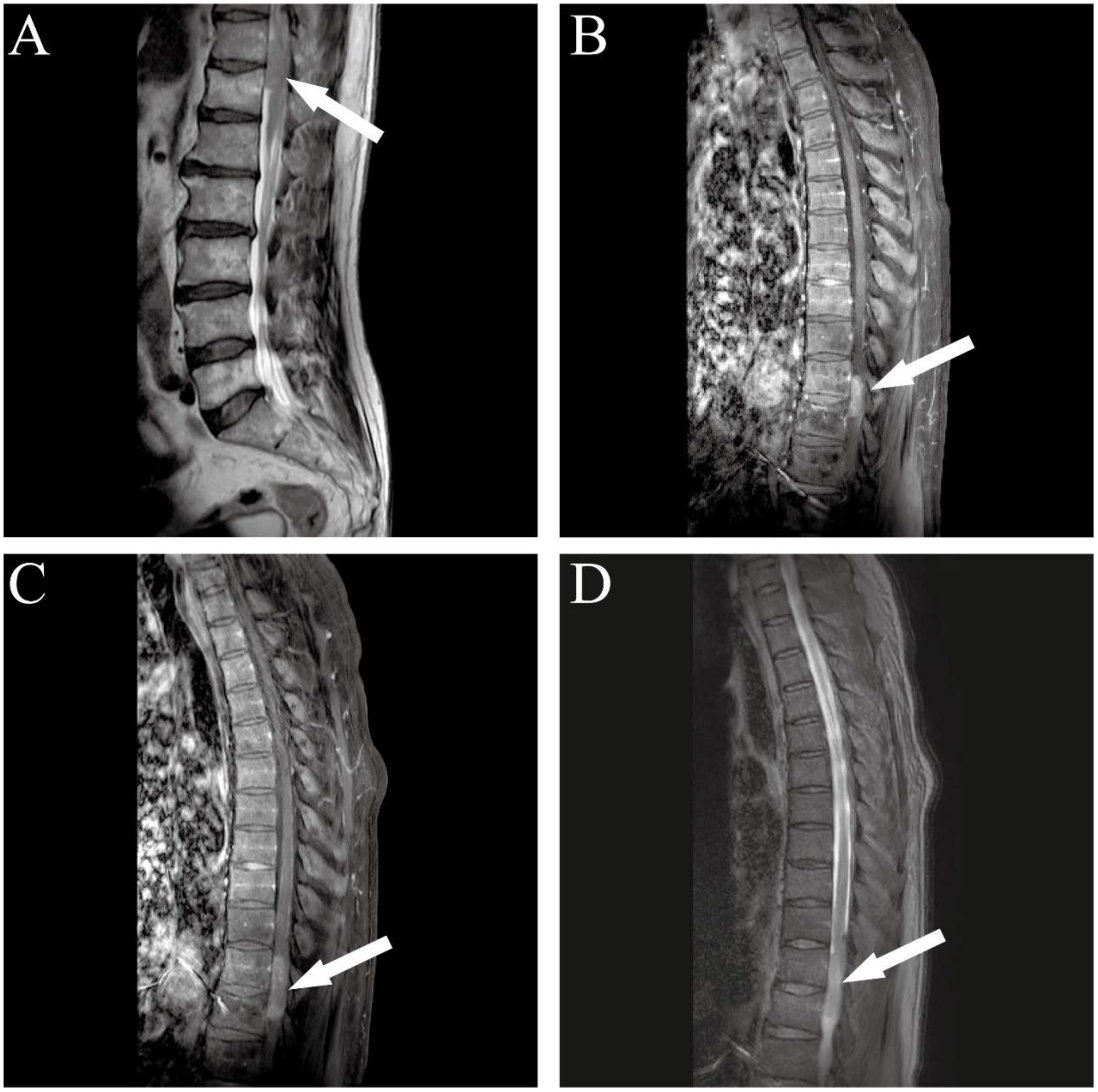
Sagittal MRI of the thoracic and lumbar spine. **(A)** T2-weighted imaging (T2WI) without contrast demonstrates an extramedullary subdural lesion at the T11–T12 spinal canal level (white arrow), exhibiting iso- to mildly hyperintense signal relative to the spinal cord. **(B)** Contrast-enhanced T1-weighted imaging (T1WI+C) reveals homogeneous enhancement of the lesion with well-defined margins, consistent with an extramedullary subdural tumor. **(C)** Follow-up T1WI+C demonstrates an increase in lesion size compared to baseline, accompanied by mildly prolonged T2 signal intensity within the spinal cord at levels T10–T12. **(D)** Corresponding fat-suppressed T2-weighted imaging (T2-FS) confirms a hyperintense intramedullary area from T10 to T12, correlating with findings on **(C)**, with effective suppression of surrounding fat signal.

Neurological examination revealed grade 3/5 proximal muscle strength (Medical Research Council [MRC] scale) in the right lower limb with marked atrophy. Deep tendon reflexes at the knees and ankles were bilaterally absent, and pathological reflexes, including the Babinski sign, were not elicited. The patient reported no family history of neurofibromatosis type 1 or 2 (NF1/NF2). Lumbar spine magnetic resonance imaging (MRI) on August 3 identified an intradural-extramedullary lesion at the T11–T12 level (**Figure 1A**). Subsequent contrast-enhanced MRI on August 4 demonstrated a well-circumscribed, heterogeneously enhancing mass measuring 3.0 × 0.9 × 1.2 cm, which compressed and displaced the spinal cord (**Figure 1B**). Radiological features were suggestive of a nerve sheath tumor. Additionally, T2-weighted images showed a patchy hyperintense signal within the T10–T12 spinal cord segments, indicative of vasogenic edema.

On August 5, 2023, serologic testing was positive for the *Treponema pallidum* particle agglutination (TPPA) assay with a toluidine red unheated serum test (TRUST) titer of 1:2, findings suggestive of syphilis. Per Centers for Disease Control and Prevention (CDC) guidelines, a definitive diagnosis of neurosyphilis requires compatible neurological manifestations corroborated by cerebrospinal fluid (CSF) analysis (e.g., a reactive CSF-VDRL test); isolated seropositivity is insufficient. Concurrently, the antinuclear antibody (ANA) titer was 1:100 (homogeneous and cytoplasmic patterns), while routine hematologic parameters were unremarkable.

A multidisciplinary team (MDT) initially recommended definitive surgical resection. However, this approach was reconsidered due to several factors: the patient lacked classical manifestations of neurosyphilis (e.g., cognitive decline, sensory ataxia, Argyll–Robertson pupils); the lesion was causing significant spinal cord compression with diminished neurologic reserve; and a lumbar puncture was deemed to carry a risk-to-benefit ratio that did not favor the procedure. Consequently, the MDT concluded that CSF evaluation was not a priority. Instead, a course of antisyphilitic therapy was initiated to mitigate potential perioperative infectious complications. Although the standard-of-care for neurosyphilis is a 10–14 day course of intravenous aqueous crystalline penicillin G, the dermatology service prescribed an intramuscular benzathine penicillin regimen (2.4 million units weekly for three weeks), which is considered inadequate for treating central nervous system infection. Based on this comprehensive assessment, surgical resection was planned following the completion of antibiotic therapy.

**Figure 2:**
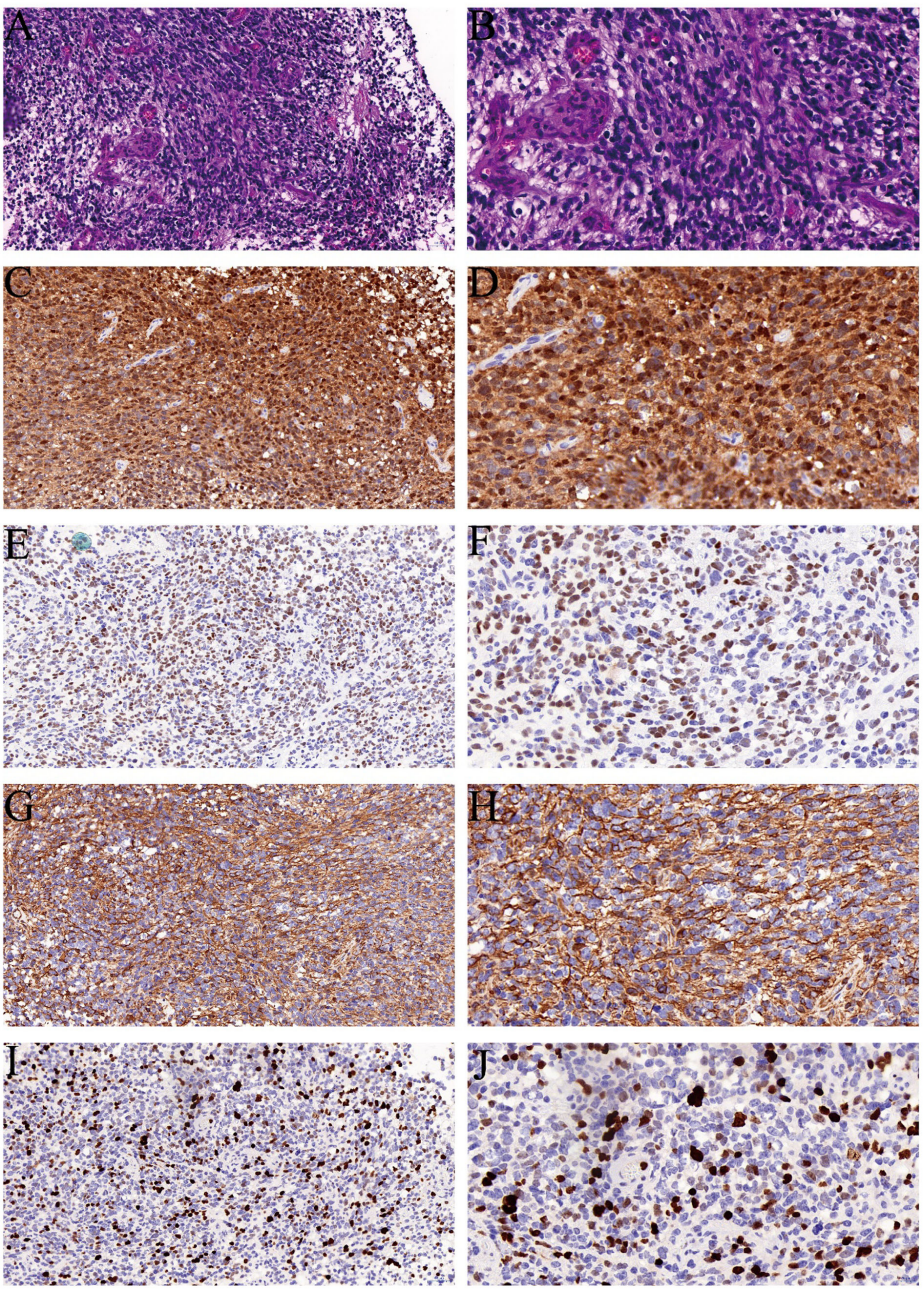
Histopathological and immunohistochemical findings of the intradural tumor. **(A, B)** Hematoxylin and eosin (H&E) staining showing tumor cells with oval and large spindle-shaped morphology; mitotic figures are visible. Magnification: **(A)** 20×, **(B)** 40×. **(C, D)** Immunohistochemistry demonstrating S-100 positivity. Original slide magnifications: **(C)** 20×, **(D)** 40×. **(E, F)** SOX-10 positive staining at 20× **(E)** and 40× **(F)** magnification. **(G, H)** Vimentin (VIM) expression indicating positivity at 20× **(G)** and 40× **(H)** magnification. **(I, J)** Ki-67 immunostaining reveals a proliferation index of approximately 50%, shown at 20× **(I)** and 40× **(J)** magnification.

On December 28, 2023, follow-up imaging upon readmission revealed tumor progression, with the mass having enlarged to 4.3 × 0.9 × 1.3 cm (**Figures 1C, D**). Subsequently, on December 29, the patient underwent a T11–T12 laminectomy for tumor resection. Intraoperatively, the tumor was observed to encase the right spinal nerve root with dense adhesions. An intracapsular piecemeal resection was performed, resulting in macroscopic residual disease (R2 resection), with a thin layer of tumor remaining adherent to the ventral spinal cord. Intraoperative frozen section analysis suggested a high-grade spindle cell neoplasm with brisk mitotic activity (>5/10 high-power fields), raising suspicion for malignancy (**Figures 2A, B**). Final histopathological examination on January 7, 2024, confirmed the diagnosis of a grade IV malignant peripheral nerve sheath tumor (MPNST) according to World Health Organization (WHO) classification. Immunohistochemistry demonstrated positivity for S-100, SOX-10, and vimentin (VIM), with loss of H3K27me3 expression and a Ki-67 proliferation index of approximately 50% (**Figures 2C-J**).

Postoperatively, the patient received high-dose intravenous methylprednisolone (480 mg/day) and prophylactic ceftriaxone (2 g daily). Postoperative inflammatory markers were monitored; on day 3, the white blood cell count was 4.73 × 10^9^/L and C-reactive protein (CRP) was 28 mg/L. The surgical wound healed without complication, and muscle strength in the right lower limb was stable at grade 3+/5. The MDT re-evaluated the indication for adjuvant radiotherapy. Given that the residual tumor was situated in close proximity to the spinal cord, the risk of radiation-induced myelopathy was deemed substantial. Therefore, after a thorough risk-benefit analysis, the decision was made to withhold adjuvant radiotherapy, with the fully informed consent of the patient and his family. The patient was discharged on postoperative day 10.

**Figure 3:**
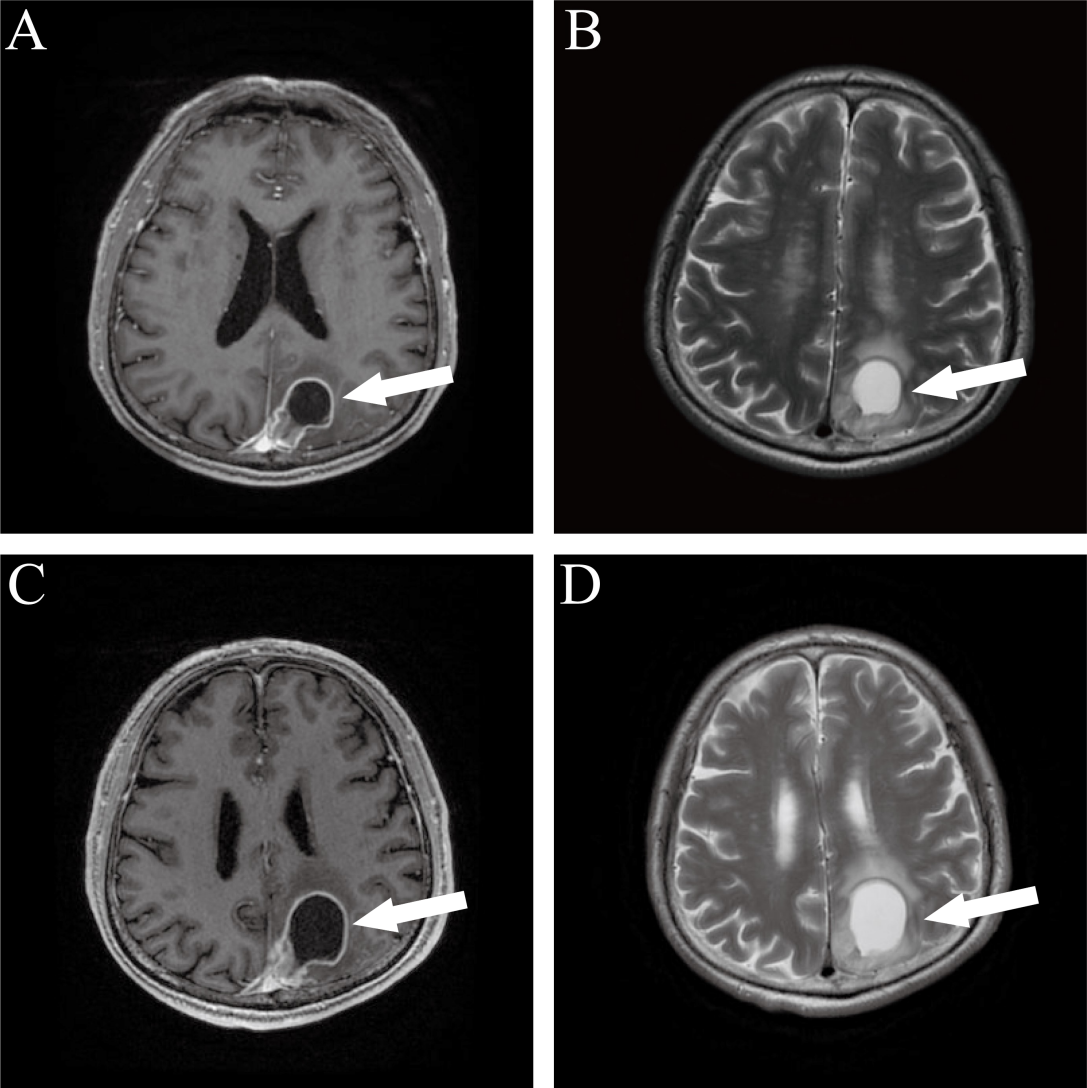
Serial brain MRI follow-up. **(A)** Contrast-enhanced axial T1-weighted imaging (T1WI+C) reveals a left parietal cystic-solid mass with ring enhancement of the cyst wall and surrounding edema. **(B)** Axial T2-weighted imaging (T2WI) shows uniform high signal intensity within the cystic region and slightly hyperintense perilesional edema. **(C, D)** Followup imaging approximately one month later. **(C)** T1WI+C demonstrates lesion enlargement compared to previous scan, with obscured boundaries adjacent to the superior sagittal sinus. **(D)** Axial T2WI reveals newly developed patchy, slightly prolonged T2 signal in the pons with ill-defined margins.

On March 6, 2024, the patient was readmitted with new-onset seizures, which had been occurring for one month and culminated in status epilepticus. Cranial MRI on February 14 had revealed a 3.7 × 2.4 cm mass in the left parietal lobe with significant perilesional edema. A follow-up contrast-enhanced MRI on March 7 showed progression of this mass to 4.6 × 3.0 cm and identified a new metastatic lesion in the pons (**Figures 3A, B**). Intracranial metastases from the primary thoracic MPNST were suspected. The antiepileptic regimen was optimized to sustained-release valproic acid (500 mg twice daily), with therapeutic serum levels maintained. Although the MDT recommended a biopsy to confirm the diagnosis, the patient’s family declined.

**Figure 4:**
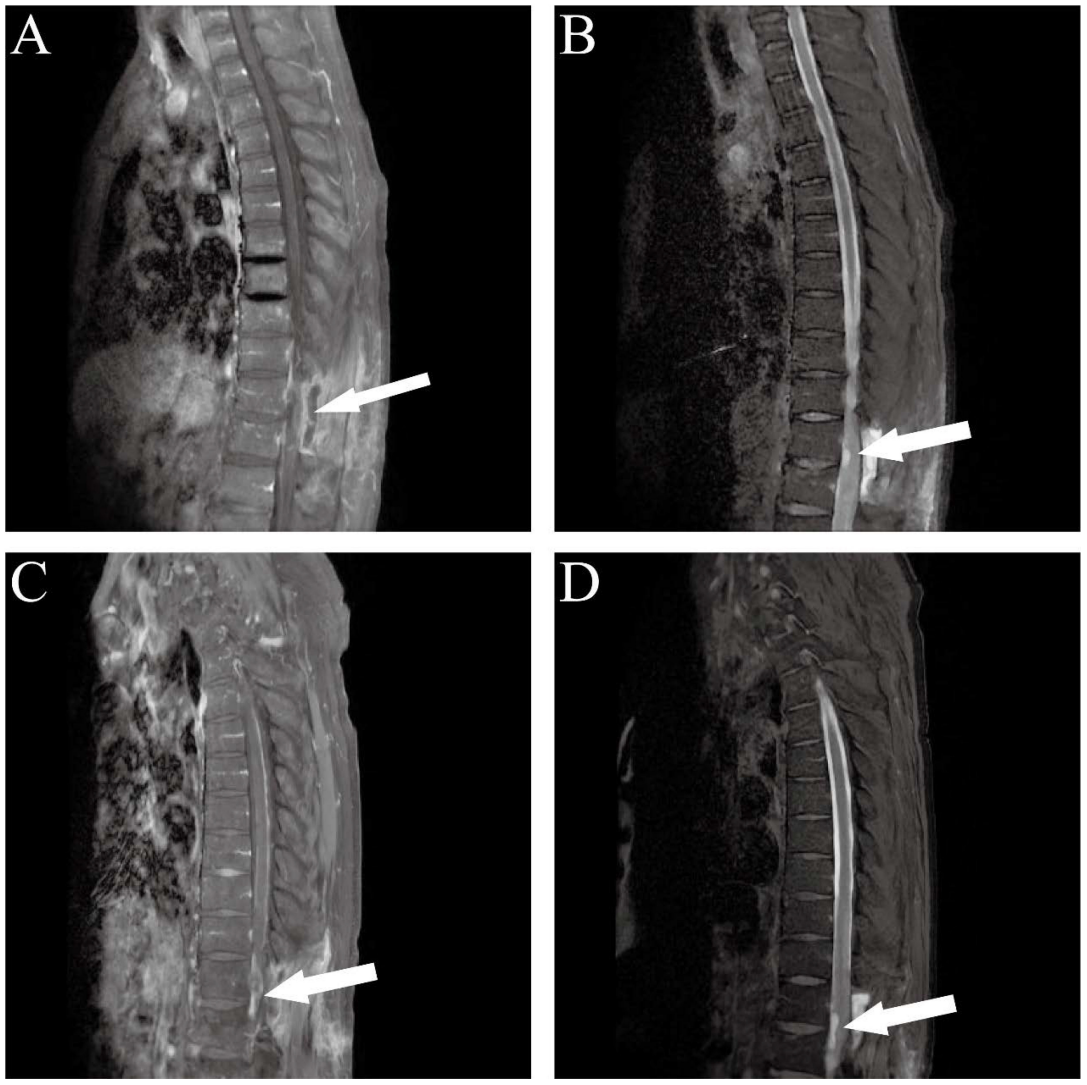
Dynamic postoperative MRI follow-up of the thoracic spine. **(A, B)** Early postoperative images. **(A)** Sagittal contrast-enhanced T1-weighted imaging (T1WI+C) showing postoperative epidural fluid collection and soft tissue swelling at the T11–T12 level. **(B)** Sagittal T2-weighted fat-suppressed imaging (T2-FS) demonstrating hyperintense fluid collection at the surgical site with surrounding inflammatory high signal. **(C, D)** Follow-up imaging approximately 1.5 months after surgery. **(C)** Sagittal T1WI+C reveals an enhancing intradural nodule at T12 level, indicative of tumor recurrence. **(D)** Sagittal T2WI demonstrates the nodule with mildly prolonged T2 signal and enlargement of the epidural linear high-signal area.

**Figure 5:**
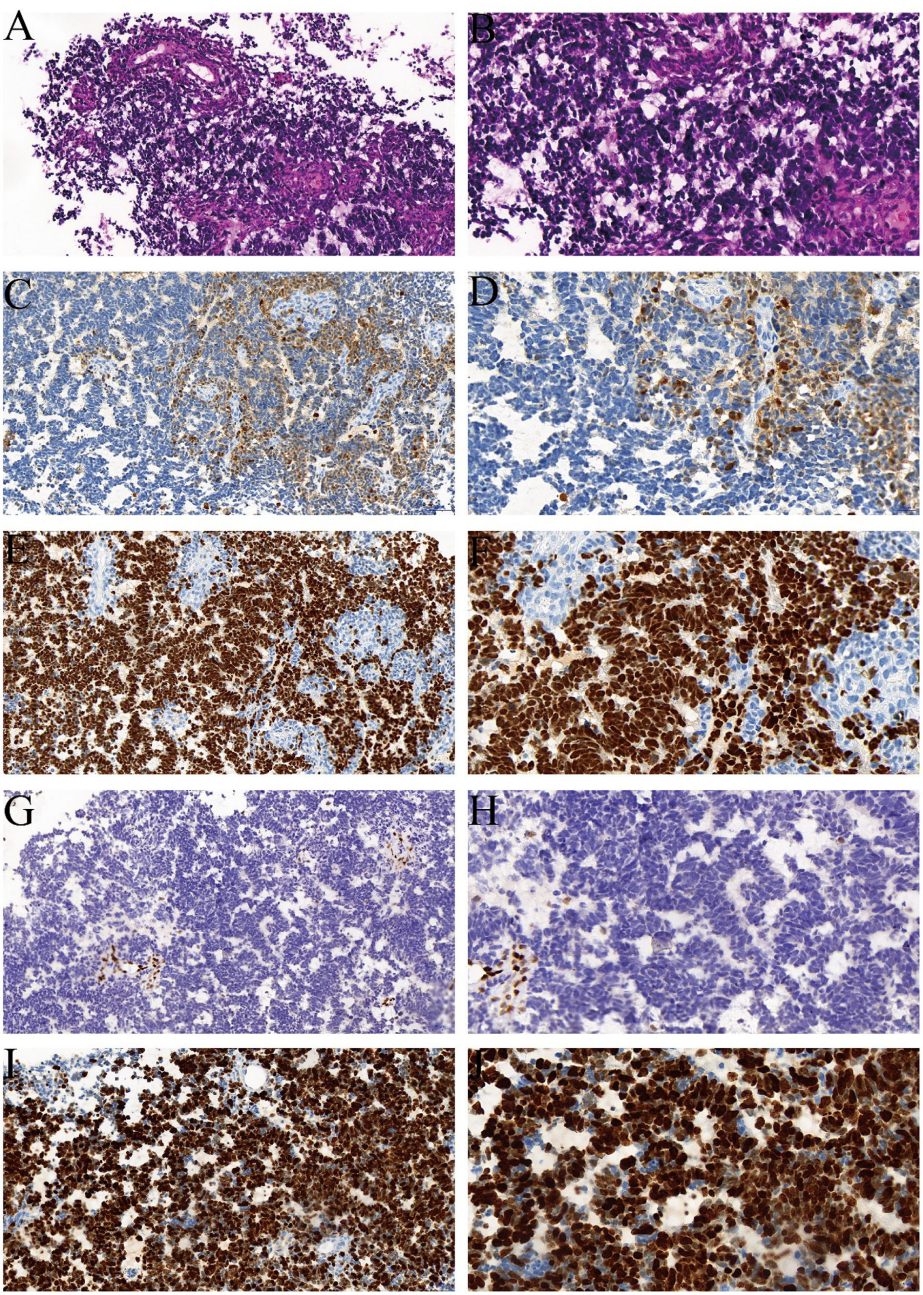
Histopathological and immunohistochemical findings of the intracranial metastatic tumor. **(A, B)** Hematoxylin and eosin (H&E) staining showing tumor cells with oval and large spindle-shaped morphology; mitotic figures are evident. Magnification: **(A)** 20×, **(B)** 40×. **(C, D)** Immunohistochemistry demonstrating S-100 positivity. Original slide magnifications: **(C)** 20×, **(D)** 40×. **(E, F)** SOX-10 positive staining at 20× **(E)** and 40× **(F)** magnification. **(G, H)** Negative H3K27me3 expression at 20× **(G)** and 40× **(H)** magnification. **(I, J)** Ki-67 immunostaining reveals a proliferation index of approximately 70%, shown at 20× **(I)** and 40× **(J)** magnification.

At the final follow-up, cranial computed tomography (CT) on March 25 revealed further progression of the left parietal mass (**Figures 3C, D**). Thoracic spine MRI on April 12 demonstrated recurrence at the primary site, with the intradural lesion at T12 enlarging to 1.5 cm and causing spinal cord compression (**Figure 4**). Despite the poor prognosis, the MDT recommended palliative debulking of the principal parietal metastasis to establish a definitive histopathological diagnosis, alleviate mass effect, and reduce seizure burden. After extensive counseling, the family elected to proceed with surgical management. Postoperative histopathology confirmed metastatic MPNST with an immunophenotype consistent with the primary tumor; however, the Ki-67 index had increased to 70%, indicating a higher proliferative activity (**Figure 5**).

**Figure 6:**
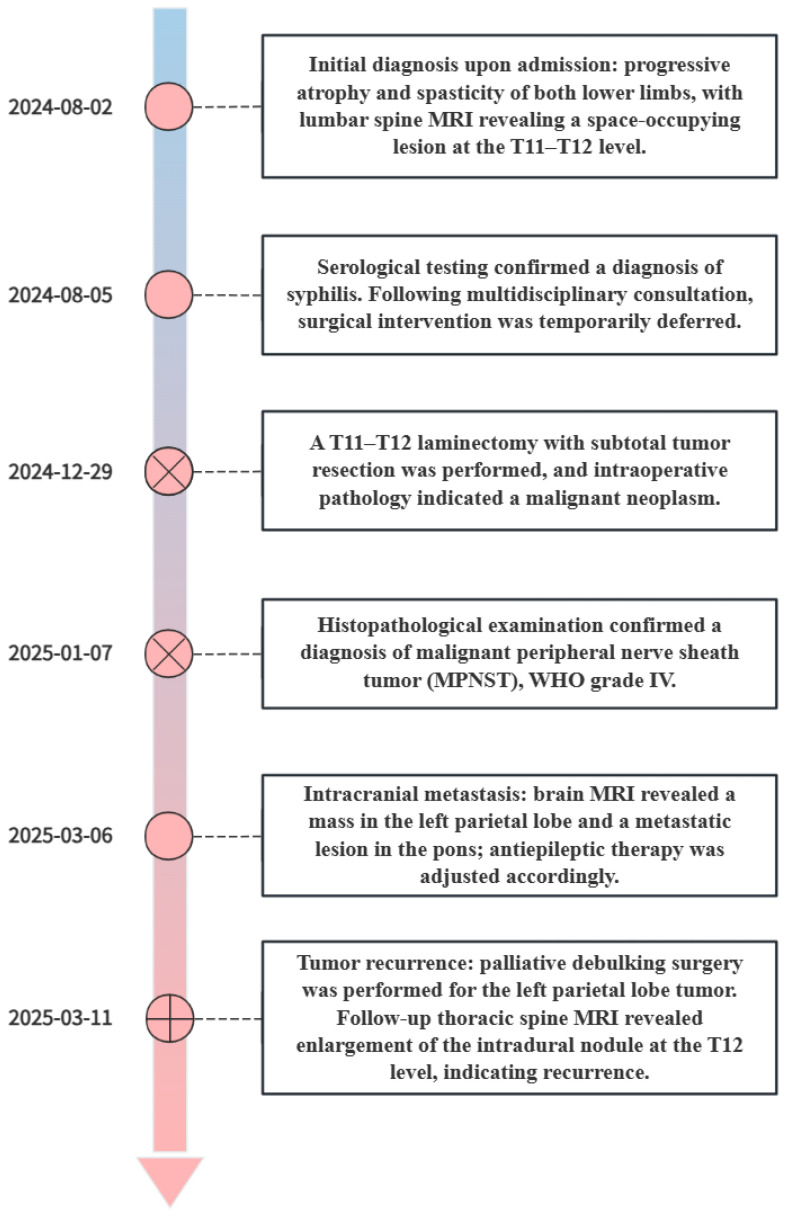
Disease course diagram.

Following this final surgery, rapid recurrence was observed within the surgical cavity (**Figure 6**). The patient’s family declined further radiotherapy and chemotherapy. The patient was transitioned to best supportive care and ultimately discharged for home hospice care. This case highlights the aggressive clinical course and high metastatic potential of spinal MPNST. Furthermore, it underscores the necessity of intensive postoperative surveillance and a coordinated multidisciplinary approach to management, providing critical insights into the therapeutic challenges posed by this rare malignancy.

## 3 Discussion

Malignant peripheral nerve sheath tumors (MPNSTs) are highly aggressive sarcomas originating from Schwann cells, constituting approximately 3%–5% of all soft tissue sarcomas (1) and are characterized by a dismal five-year survival rate. Approximately 4%–5% of cases are associated with Neurofibromatosis type 1 (NF1), while the remainder arise sporadically or secondary to prior radiotherapy. The absence of specific molecular biomarkers renders early diagnosis a significant clinical challenge. Primary sporadic intradural MPNSTs are exceedingly rare, with the thoracic spine being the most frequently reported site of origin (2, 3).

Here, we present the case of a 64-year-old patient with progressive lower-limb deficits, where the aggressive nature of the spinal lesion necessitated a complex differential diagnosis, including neurosyphilis. Initial serology showed positive Treponema pallidum particle agglutination (TPPA) with a low titer (1:2) on the toluidine red unheated serum test (TRUST). The patient presented with progressive lower-limb sensorimotor deficits and concomitant muscle atrophy. Given the considerable phenotypic overlap with neurosyphilis, particularly a tabes dorsalis–like presentation, it was considered a primary differential diagnosis. The diagnosis of neurosyphilis is established by integrating neurological manifestations with serological and cerebrospinal fluid (CSF) analyses; the latter typically reveals pleocytosis, elevated protein levels, or a reactive CSF-Venereal Disease Research Laboratory (VDRL) test. However, in this patient, the atypical clinical and imaging findings, coupled with inconclusive serology, did not substantiate a diagnosis of neurosyphilis, prompting a decision to defer surgical intervention. In cases where the CSF-VDRL is nonreactive despite high clinical suspicion, CSF-Fluorescent Treponemal Antibody Absorption (FTA-ABS) or CSF-TPPA assays can enhance diagnostic sensitivity (4). The standard-of-care treatment is a 10–14 day course of intravenous aqueous crystalline penicillin G (5). Intramuscularly administered benzathine penicillin fails to achieve therapeutic bactericidal concentrations within the central nervous system (CNS) and is therefore contraindicated as monotherapy; its use is restricted to adjunctive therapy following the completion of a standard intravenous regimen (5).

Radiologically, spinal neurosyphilis is typically characterized by long-segment T2 hyperintensity, predominantly affecting the dorsal columns, accompanied by myelitis-like changes and patchy enhancement—features notably absent in our patient. In rare instances, a syphilitic gumma may present as a discrete, mass-like lesion. Appropriate penicillin therapy often leads to radiologic improvement or resolution (6). In this patient, however, CSF analysis provided no corroborating evidence, and the imaging features were atypical for neurosyphilis, collectively undermining the evidentiary basis for this diagnosis. Furthermore, the administration of benzathine penicillin, an inappropriate regimen for CNS disease, precluded a definitive assessment of its therapeutic effect on either symptom progression or tumor kinetics. The decision to delay surgery, reached after multidisciplinary team (MDT) deliberation to mitigate perceived infectious risk, proved consequential. Despite this precaution, the patient’s neurological symptoms progressively worsened in parallel with tumor enlargement on imaging. Postoperative histopathology ultimately confirmed a high-grade MPNST, establishing that the clinical deterioration was driven by neoplastic progression rather than an infectious process.

This case underscores a critical clinical principle: in the presence of serological abnormalities, if imaging and clinical data strongly indicate a neoplastic process—such as a focal mass causing progressive spinal cord compression—surgical intervention should not be unduly delayed on the basis of seropositivity alone. Concurrently, CSF analysis and other differential evaluations must be expedited. If an infectious etiology is substantiated, standardized intravenous antimicrobial therapy can be coordinated with infectious disease specialists in the perioperative period, carefully balancing infection management against the risk of irreversible neurological decline from tumor progression. Therefore, the optimal clinical pathway for a progressive mass lesion should prioritize timely surgical intervention while simultaneously expediting definitive CSF-based diagnostics. Should evidence of a concomitant infection emerge, perioperative and long-term management strategies must then be adjusted accordingly.

A comparison with the limited number of analogous cases in the literature underscores the distinctive clinical trajectory and therapeutic impasse associated with this tumor. As summarized in **Table 1**, the collective data from these cases reveal sobering commonalities: despite heterogeneity in the primary site—ranging from the cauda equina and lumbar spine to the thoracic segment in the present case—and in age at diagnosis, the prognosis becomes uniformly dismal following intracranial metastasis, with survival periods typically lasting only months. This prognostic pattern starkly illustrates the limitations of current therapeutic modalities against the tumor’s intrinsic biological behavior. Notably, the interval from initial surgery to the detection of intracranial metastasis in our patient (≈2 months) was substantially shorter than that reported in most published instances. This exceptional clinical aggression is consistent with the tumor’s high proliferative index (Ki-67 50%) and the R2 resection status, suggesting early dissemination of gross or microscopic residual disease along the neuraxis, potentially via perineural spread or CSF-mediated seeding.

**Table 1:** Postoperative intracranial metastasis from spinal/intradural MPNST: literature cases and the index case.

Author (Year)	References	Age/Sex	Primary Location	NF1 Status	Primary-tumor treatment (surgery/RT/CT)	Time to intracranial mets (mo)	Intracranial metastasis type	Treatment of metastasis	Outcome (Survival)
Xu et al. (2012)	(7)	8/M	Cauda equina L3–L5	Sporadic	Initial GTR → two re-resections; spinal RT 40.5 Gy	14	Parenchymal (right cerebellar peduncle)	Cranial RT 30 Gy	Died 16 mo postop
Park et al. (2013)	(8)	18/M	Lumbar L2–L3 (to psoas)	Sporadic	GTR (hemilaminectomy); no RT	6	Parenchymal (pons & cerebellum)	Chemotherapy incl. intrathecal (ineffective)	Died ~10 mo postop (≈4 mo after mets)
Stark & Mehdorn (2013)	(9)	56/F	Sacral S1 nerve root	Sporadic	Resection; no adjuvant RT *(prior lymphoma RT)*	19	Parenchymal (brainstem) + LM (lumbar & intracranial)	Palliative WBRT	Died 5 mo after mets *(24 mo total)*
Li et al. (2014)	(10)	33/F	T12–L1	Sporadic	Two partial resections; RT 28 Gy/19 fx	0.5 *(after 2nd op)*	Parenchymal (left midbrain) + LM (spinal dissemination)	VPS; no specific anti-met tx reported	Died 29 mo after initial dx
Baharvahdat et al. (2018)	(11)	3/F	Cervical C1–T1	Sporadic	Subtotal resection; no RT/CT	0.3 *(9 days postop)*	Parenchymal (brainstem & temporal) + LM	EVD; no RT/CT	Died shortly after (hydrocephalus)
Present Case	64/M	64/M	Thoracic T11–T12 (intradural, extramedullary subdural)	Sporadic (no NF1/NF2 history)	Subtotal resection via T11–T12 laminectomy (intracapsular piecemeal; R2); no adjuvant RT/CT	1.5–2	Parenchymal (left parietal; pons)	Palliative craniotomy (parietal debulking); no RT/CT	Alive

mo, months; wks, weeks. Conversions used in text: 9 days ≈ 0.3 mo; 2 wks ≈ 0.5 mo. RT/CT, radiotherapy/chemotherapy; WBRT, whole-brain radiotherapy; craniospinal RT (CSI), craniospinal radiotherapy/irradiation. GTR, gross total resection (complete macroscopic tumor removal); debulking/partial resection: subtotal tumor removal. R2: macroscopically positive margin with gross residual disease (subtotal/incomplete resection). postop, postoperative; dx, diagnosis; f/u, follow-up. LM, leptomeningeal metastasis; parenchymal, brain parenchymal metastasis. EVD, external ventricular drainage; VPS, ventriculoperitoneal shunt. intrathecal, drug administration into the cerebrospinal fluid; palliative: treatment aimed at symptom relief/quality of life rather than cure.

Intracranial metastasis of MPNST is a hallmark of advanced disease, associated with a median survival of less than five months. For primary spinal MPNSTs, the extent of surgical resection is a key determinant of prognosis. In the present case, the patient developed intracranial metastasis two months post-resection of the thoracic MPNST and experienced local recurrence within three months, indicating extremely rapid disease progression. This aggressive course is likely attributable to microscopic residual tumor following an R2-level subtotal resection, which may have seeded early micrometastases. Moreover, histopathology revealed a Ki-67 proliferation index of 50%, far exceeding the poor-prognosis threshold of ≥20%, and scattered positivity for H3K27me3. These findings suggest the presence of epigenetic heterogeneity that may be driving the tumor’s aggressive biological behavior (12).

In this case, the patient developed an intracranial metastatic lesion following resection of the intradural MPNST, with imaging revealing a cystic-solid mass. Based on metastatic pathways and anatomical characteristics, intracranial dissemination of MPNST can be broadly classified into two patterns: brain parenchymal metastasis (BM) and leptomeningeal metastasis (LM). Distinguishing between these patterns is crucial for determining appropriate therapy. Here, the clinical presentation favored BM, but LM could not be definitively ruled out. A definitive distinction would directly guide therapy: isolated BM would justify surgical resection or stereotactic radiosurgery (SRS), whereas LM would indicate whole-brain radiotherapy (WBRT) or intrathecal chemotherapy. The family declined invasive diagnostics, including CSF cytology or targeted biopsy, precluding a definitive diagnosis. Consequently, management defaulted to conservative WBRT, highlighting the challenge of individualizing treatment under diagnostic uncertainty.

The differentiation of intracranial dissemination patterns—specifically, distinguishing isolated BM from concomitant LM—is of paramount clinical importance, as it dictates the subsequent therapeutic strategy. While parenchymal lesions may be amenable to local control via palliative surgery or SRS, as was performed for the parietal lesion in this case, the presence of diffuse LM necessitates a paradigm shift toward systemic, non-focal modalities such as WBRT or intrathecal chemotherapy. In this instance, abnormal signal within the pons precluded the definitive exclusion of LM. Consequently, the family’s refusal to consent to additional invasive diagnostic procedures mandated a conservative management approach based on limited imaging data, highlighting the clinical challenge of balancing diagnostic uncertainty with patient preferences and therapeutic urgency.

Collectively, this case and the existing literature underscore that high-grade, subtotally resected spinal MPNSTs are associated with a substantial risk of rapid intracranial dissemination, necessitating a high index of clinical suspicion and rigorous surveillance. Furthermore, a precise characterization of intracranial lesions is imperative, given that the therapeutic paradigms for BM and LM differ fundamentally. Ultimately, significant prognostic improvements for these patients will likely depend on the development of systemic agents with enhanced penetration of the blood-brain and blood-CSF barriers.

Importantly, intracranial metastases from spinal MPNSTs may present as atypical cystic-solid lesions, rather than exhibiting the classic imaging features of LM. In patients with a history of spinal surgery and no evidence of extracranial metastases, LM should be considered with a high index of suspicion. For such cases, a comprehensive assessment integrating primary tumor location, metastatic pathway, and multimodal diagnostic tools—such as CSF cytology and targeted biopsy—is essential to avoid misinterpretation based solely on imaging.

Although computed tomography (CT) and magnetic resonance imaging (MRI) are the primary modalities for evaluating MPNSTs, they have significant limitations in differentiating between benign and malignant lesions. A key diagnostic challenge lies in distinguishing MPNSTs from benign nerve sheath tumors, which typically present as well-circumscribed lesions under 5 cm in diameter, often exhibiting characteristic features such as the “target sign” and “fat split sign.” MPNST must also be differentiated from other soft tissue sarcomas, such as alveolar soft part sarcoma and synovial sarcoma. However, the MRI features of MPNSTs often overlap considerably with those of benign schwannomas, further complicating an accurate diagnosis (13–15).

In this patient, the initial thoracic MRI revealed a 3.0 cm subdural extramedullary nodule with slightly hyperintense signals on both T1- and T2-weighted imaging, along with marked heterogeneous enhancement—features suggestive of a nerve sheath tumor. However, the poorly defined lesion margins and associated intramedullary spinal cord edema (T10–T12 high signal intensity) were indicative of malignant potential. Within three months postoperatively, follow-up imaging showed rapid tumor growth to 4.3 cm with increased enhancement heterogeneity. Notably, the scattered positivity for H3K27me3 may reflect subclonal heterogeneity within the tumor. This epigenetic imbalance could contribute to malignant progression by activating oncogenes such as IGF2 and PAX2, ultimately driving high proliferative activity (Ki-67 index up to 70%) and aggressive clinical behavior.

While high-grade MPNSTs tend to exhibit greater contrast enhancement and larger tumor diameters on MRI, their morphological features—such as ill-defined margins and areas of cystic degeneration or necrosis—substantially overlap with those of lower-grade tumors. Therefore, imaging cannot supplant histopathological grading. In this case, the intracranial metastatic lesion demonstrated a “target ring sign” with surrounding edema, while the newly developed pontine nodule was consistent with hematogenous dissemination. The cystic and necrotic characteristics aligned with known patterns of aberrant angiogenesis in metastatic MPNST but also necessitated differentiation from vascular lesions related to infectious etiologies (16, 17).

The pathological diagnosis of MPNST requires the integration of histomorphology, immunophenotyping, and molecular profiling. Differentiating it from benign schwannomas, undifferentiated sarcomas, and melanomas is particularly challenging. Histologically, MPNST is characterized by hypercellular spindle cell proliferation with marked nuclear atypia, frequent necrosis, and endothelial proliferation. Immunohistochemically, S-100 and SOX-10 positivity supports a nerve sheath origin. However, caution is warranted, as some NF1-associated cases may aberrantly express Melan-A or MITF. Negative staining for HMB45, PNL2, and the absence of a BRAF V600E mutation help exclude melanoma. Loss of H3K27me3 expression is a valuable molecular marker for distinguishing MPNST from undifferentiated sarcomas (18–20). Interestingly, in our case, the primary lesion showed scattered positivity for H3K27me3, whereas the metastatic lesion was completely negative, suggesting that progressive epigenetic silencing may correlate with increased tumor aggressiveness.

Of note, the initial spinal lesion was radiologically misdiagnosed as a benign schwannoma. The presence of atypical cells on intraoperative frozen section prompted a diagnostic revision, underscoring the critical role of intraoperative or preoperative pathological verification. Ultimately, histopathological confirmation—such as that obtained following resection of the parietal metastasis—remains the gold standard for definitively excluding primary intracranial neoplasms.

There is currently no standardized treatment protocol for primary sporadic intradural MPNST, although surgical resection remains the cornerstone of management (21). The therapeutic role of radiotherapy for spinal cord/intradural MPNST remains a subject of debate, with no definitive consensus on its optimal use. This uncertainty stems primarily from the profound rarity and aggressive nature of the disease, coupled with a paucity of systematic therapeutic evidence. The majority of available evidence is derived from retrospective cohorts and case series, which consistently establish surgical resection as the cornerstone of management (7). However, in intradural locations—particularly within the cauda equina and perimedullary regions—wide en bloc resection is often infeasible owing to anatomical constraints and the imperative to preserve neural function. Consequently, adjuvant radiotherapy is frequently considered to enhance local control (LC), although its impact on overall survival (OS) remains equivocal (22).

Postoperative radiotherapy is thought to enhance LC, particularly in cases involving large tumors (e.g., >5 cm), high-grade histology, or positive margins following R1/R2 resection. For instance, one analysis of 350 patients demonstrated that the omission of adjuvant radiotherapy was associated with a significantly elevated 5-year risk of local failure (risk ratio 4.50). Similarly, single-center retrospective series suggest that perioperative radiotherapy may prolong both LC and OS. Nevertheless, a durable OS advantage has not been uniformly demonstrated; for example, analyses of the SEER spine MPNST dataset have reported an association between receipt of radiotherapy and worse survival, likely reflecting confounding by indication (22). Collectively, these findings underscore the necessity for cautious interpretation of retrospective data and highlight the importance of individualized therapeutic decision-making. Notably, the current body of evidence is exclusively retrospective; prospective randomized trials to provide definitive guidance are lacking.

The application of radiotherapy in the intradural setting is fundamentally constrained by the radiation tolerance of the spinal cord. Under conventional fractionation, the accepted spinal cord dose limit is typically ≤54 Gy, whereas standard postoperative doses for sarcoma range from 60–66 Gy. This therapeutic “dose gap” substantially compromises the potential for achieving effective local control (23). To bridge this dose gap, contemporary high-precision modalities—such as proton therapy (median prescription 64 GyRBE) and carbon-ion therapy—are increasingly being utilized. The distinct physical and biological properties of these modalities permit enhanced target volume coverage while simultaneously minimizing radiation dose to the spinal cord (24). Preliminary data suggest these approaches have an acceptable toxicity profile and yield encouraging rates of LC. Nevertheless, their long-term effectiveness requires rigorous validation through larger cohort studies. Particularly for patients with NF1-associated disease or a history of prior irradiation, the substantial risks of re-irradiation—including second malignant neoplasms and severe treatment-related complications—mandate a rigorous, multidisciplinary, and individualized assessment of the risk-benefit ratio before finalizing treatment strategies (24).

The traditional Enneking-appropriate (EA) resection, which emphasizes en bloc excision with tumor-free margins, was once considered the optimal surgical strategy for spinal tumors. However, multicenter studies have shown no significant difference in local recurrence rates between EA (44%) and Enneking-inappropriate (EI) resections (33%) (25, 26). This discrepancy is largely attributed to the tumor’s tendency for skip metastasis along nerve roots and its tight adherence to critical intradural structures, which often necessitates marginal or even intralesional resection (27).

In this case, local recurrence occurred within three months postoperatively, suggesting that conventional en bloc resection may be insufficient to eliminate microscopic perineural spread. Intraoperative radiotherapy (IORT) or more aggressive surgical strategies—such as sacrificing involved nerve roots—may be necessary to improve local control (28). Moreover, limitations in pathological assessment, including interobserver variability in mitotic index interpretation and sampling bias during intraoperative frozen section, can affect surgical decision-making and increase the risk of neural injury or early recurrence (29).

Although surgery remains the primary curative modality, the clinical benefit of adjuvant radiotherapy and chemotherapy remains controversial. Chemotherapeutic regimens, such as anthracycline-based combinations with ifosfamide, are associated with significant toxicity and rapid development of resistance, offering limited survival advantage (30).

Notably, in this case, subtotal resection was performed due to dense adhesion between the tumor and spinal nerve root. This likely facilitated intraneural dissemination via perineural sleeves, potentially explaining the rare and rapid onset of leptomeningeal metastasis observed shortly after surgery. These findings underscore the critical importance of early radical resection in preventing recurrence and distant spread of intradural MPNST. Residual tumor may disseminate along the neuroaxis, resulting in atypical intracranial metastases, particularly of the leptomeningeal subtype. The management of advanced-stage MPNST is predicated on a palliative and supportive care framework, prioritizing symptom alleviation, the preservation of quality of life, and adherence to patient and family preferences.

Accurate diagnosis of spinal MPNST requires integration of tumor location, imaging, and pathology. Future efforts should focus on combining neurophysiological monitoring with molecular risk profiling to guide surgical decisions and personalize adjuvant therapy, aiming to improve outcomes beyond the current survival plateau.

## 4 Conclusion

This case highlights the rare occurrence of early intracranial metastasis in intradural MPNST, emphasizing its aggressive clinical behavior. Rapid cranial dissemination following subtotal resection suggests a potential role of early microscopic spread along neural pathways. Given the limitations of imaging and intraoperative pathology in detecting early metastasis, close postoperative surveillance is critical. Integrating molecular risk assessment with individualized surgical planning may aid in reducing early intracranial progression and improving outcomes in this highly invasive tumor.

## Data Availability

The raw data supporting the conclusions of this article will be made available by the authors, without undue reservation.
